# Juxtamembrane 2 mimic peptide competitively inhibits mitochondrial trafficking and activates ROS-mediated apoptosis pathway to exert anti-tumor effects

**DOI:** 10.1038/s41419-022-04639-6

**Published:** 2022-03-24

**Authors:** Dan He, Zhijie Ma, Ke Xue, Haiyan Li

**Affiliations:** 1 School of Biomedical Engineering, Shanghai Jiao Tong University, 1954 Huashan Road, Shanghai, 200030 China; 2 Department of Plastic and Reconstructive Surgery, Shanghai Ninth People’s Hospital, Shanghai Jiao Tong University School of Medicine, 639 Zhi Zao Ju Rd, Shanghai, 200011 China; 3 Chemical and Environmental Engineering, School of Engineering, RMIT University, 124 La Trobe Stø, Melbourne, VIC 3000 Australia

**Keywords:** Biomedical materials, Breast cancer, Apoptosis, Peptide nucleic acid oligo, Chemotherapy

## Abstract

Our previous study demonstrates that a juxtamembrane 2 (JM2) mimic peptide can inhibit proliferation and induce apoptosis of tumor cells. However, the mechanism remains unclear. In this study, JM2 is found to suppress the growth of 4T1 breast tumors by inducing apoptosis and inhibiting the proliferation of 4T1 tumor cells. Further study indicates that JM2 can stimulate the mitochondria to gather near the microtubule-organizing center of tumor cells and subsequently induce ROS-induced ROS release responses, which results in mitochondrial dysfunction and mitochondria-mediated apoptosis. In addition, JM2 can arrest cell cycle in S phase by regulating the expression of cell cycle-related proteins and consequently inhibit proliferation of tumor cells. Then, a previously designed JM2 grafted hyaluronic acid (HA) injectable hydrogel system (HA-JM2) is injected in a breast tumor-resected model and the HA-JM2 hydrogel can inhibit the malignant proliferation of residual tumor cells and suppress the breast tumor recurrence. These findings not only confirm the application potentials of JM2 in anti-tumor therapy and tumor post-surgery treatments but also provide greater understanding on the mechanisms by which JM2 inhibits tumor growth.

## Introduction

Suppression of tumor growth plays an important role in tumor therapy and tumor post-surgery treatment. Up to now, common treatment modalities for tumor therapy include chemotherapy [1], radiation therapy [2], surgery [3], thermotherapy [4], immunotherapy [5], or a combination of these therapies [6]. Once the tumor has to be removed, surgery is the most common intervention. However, residual tumor cells at the surgical margin are hard to be avoided and local tumor recurrence happens at a very high rate [7]. Clinically, chemotherapy drugs are used to eradicate cancer cells or stop spreading of cancer cells to prevent tumor growth or recurrence, but side effects such as toxicity to healthy tissues or cells are inevitable [8, 9]. Therefore, it is an urgent need to explore new anti-tumor therapeutic drugs for tumor therapy and tumor post-surgery treatment.

In our previous study, we found that a connexin 43 (Cx43) mimic peptide, juxtamembrane 2 (JM2), could induce apoptosis of melanoma cells (B16F10). However, the full mechanisms for JM2 inducing apoptosis and inhibiting proliferation of tumor cells and subsequently suppressing tumor growth remain unclear. JM2 is a peptide that mimics the amino acid sequence of juxtamembrane region of Cx43 molecular and contains the amino acid sequence of the microtubule-binding domain [10]. Recently, studies have shown that GJA1-20k, a fragment corresponding to the C-terminal tail of Cx43, could promote microtubule-based mitochondrial transport and the integrity of the mitochondrial network, thereby preventing the organelle network from collapsing when the cells were exposed to oxidative stress [11]. It is worth noting that the microtubule-binding domain in GJA1-20k plays an important role in promoting mitochondrial transport and maintaining mitochondrial positioning in the periphery. Since JM2 mimic peptide also contains the amino acid sequence of microtubule-binding domain, it can be hypothesized that JM2 mimic peptide can compete with mitochondria to bind to microtubules, preventing the transport of mitochondria through the microtubules to the periphery of the cells.

It is well known that mitochondria play a vital role in cell survival and apoptosis since the basic metabolic processes occur in it [12]. Reactive oxygen species (ROS), which is mainly produced in mitochondria, is a highly active molecule that affects intracellular balance and plays a key role in tumorigenesis [13]. Generation of ROS can stimulate the opening of mitochondrial permeability transition pores, which further stimulates the release of ROS and finally cause the ROS-induced ROS release (RIRR) response, which may consequently cause the damage of mitochondria and cells [14]. Studies have reported that excessive ROS levels can induce tumor cell apoptosis [15]. Thus, the production of excessive ROS has been reported to be related to anti-tumor process and more and more anti-tumor drugs aiming at increasing the ROS level in tumor cells are under development [16, 17]. In addition, it has been reported that accumulation of mitochondria in cells can increase intracellular ROS levels. Studies have shown that blocking a pathway related to mitochondrial localization can induce mitochondrial aggregation near the microtubule-organizing center. The amplification of ROS signal was observed in the aggregated mitochondrial network structure, which resembled the RIRR response [18]. Thus, increasing ROS level in tumor cells by inducing accumulation of mitochondria is a very effective ROS-mediated method for inhibiting tumor growth.

In this study, molecular signaling pathway of tumor cell apoptosis induced by JM2 was studied. The inhibitory effects of JM2 on tumor growth, proliferation, migration, and invasion of B16F10 and 4T1 cells were firstly confirmed. To elucidate the mechanism, the intracellular localization of JM2 and the distribution of intracellular mitochondria were observed. Subsequently, intracellular ROS levels and the expressions of mitochondrial apoptosis pathway-related proteins and cell cycle-related proteins were detected. Finally, in order to ensure the application of JM2 in tumor post-surgery treatments, an JM2 grafted hyaluronic acid (HA) injectable hydrogel system (HA-JM2) reported in our previous article was applied to a breast tumor resection model.

## Results

### JM2 inhibited the growth of breast tumor in vivo

**Fig. 1:**
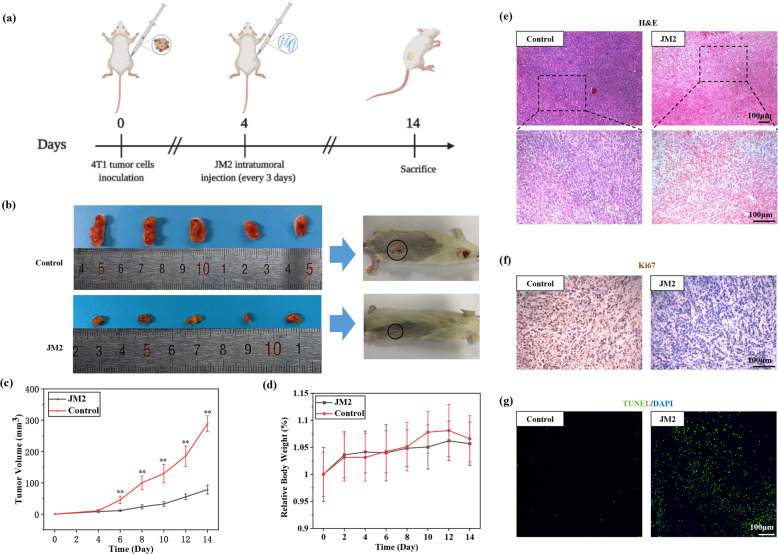
JM2 inhibited tumor growth in vivo. **a** Schematic illustration of the in vivo anti-tumor experimental protocol. **b** Representative images of tumors in Control and JM2 groups at day 14. **c** Tumor volume at each time interval measured during treatment. **d** Body weight curve of mice monitored during treatment. **e** H&E staining of tumors obtained from Control and JM2 groups at day 14. **f** Ki67 immunohistochemical staining (brown) of tumors obtained from Control and JM2 groups at day 14. **g** TUNEL staining (green) and DAPI staining (blue) of tumors obtained from Control and JM2 groups at day 14.

Figure 1a illustrates the experiment protocols. The photos of excised tumors are shown in Fig. 1b, which indicates that tumors treated with JM2 were much smaller than the ones treated with PBS (control group). On day 14, the average tumor volume in the PBS-treated group (control) was about three times larger than that in the JM2 group (Fig. 1c). To assess the potential toxicity of JM2, the body weight of the mice was constantly monitored. Figure 1d shows that the body weight of JM2-treated mice had no significant loss as compared with that of PBS-treated mice in control group, indicating the low toxicity of the JM2 in vivo.

H&E assay further demonstrated that JM2 treatment resulted in loose tissue structure and caused a distinct drop in the number of tumor cells with extensive nuclear shrinkage and fragmentation (Fig. 1e), which led to significant inhibition of the tumor growth. In addition, there were more Ki67-positive cells (brown color) in tumor tissue treated with PBS (control group) as compared with those in tumor tissue treated with JM2 (Fig. 1f). Furthermore, there were more apoptotic cells (green) in tumor tissue treated with JM2 as compared with those in tumor tissue treated with PBS (control group) (Fig. 1g). All of these results further confirmed that JM2 could remarkably inhibit the growth of breast tumors by inhibiting proliferation and inducing apoptosis of tumor cells in vivo.

### JM2 inhibited proliferation of B16F10 and 4T1 tumor cells in vitro

**Fig. 2:**
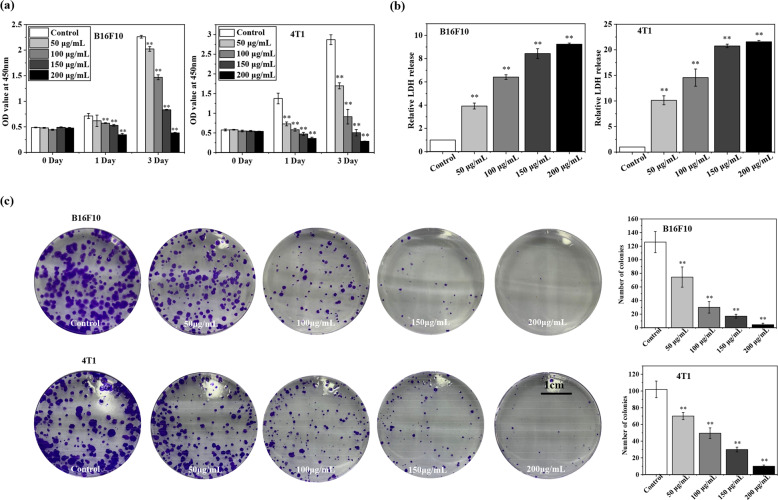
JM2 inhibited proliferation of tumor cells. **a** Cell proliferation evaluation of B16F10 and 4T1 cells cultured with JM2 solutions with different concentrations. **b** Lactate dehydrogenase analysis of B16F10 and 4T1 cells cultured with JM2 solutions with different concentrations. **c** Colony formation of B16F10 and 4T1 cells cultured with JM2 solutions with different concentrations. * *p* < 0.05 and ** *p* < 0.01 when the data was compared with the Control group.

It can be seen from Fig. 2a that JM2 exerted a significant inhibition on cell proliferation of B16F10 and 4T1 cells in a dose‐dependent as well as time‐dependent manner. Quantitative analysis of cytotoxicity can be achieved by detecting the activity of LDH released into the culture medium from the ruptured cells. The results in Fig. 2b show that B16F10 cells and 4T1 cells cultured with JM2 solutions released more LDH in the extracellular environment as compared to the control group. It can be seen from Fig. 2c that, compared to the control group without the addition of JM2, the higher the concentration of the JM2 solution, the fewer colonies of B16F10 or 4T1 cells formed in the plate. It can be concluded that JM2 could significantly suppress the colony formation ability of B16F10 and 4T1 cells in a dose-dependent manner.

### JM2 suppressed the migration and invasion of B16F10 and 4T1 tumor cells

**Fig. 3:**
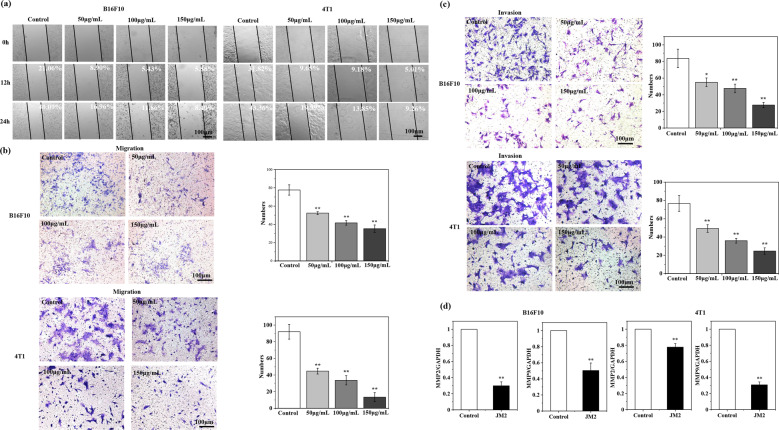
JM2 inhibited migration of tumor cells. **a** In vitro wound healing of B16F10 and 4T1 cells cultured with JM2 solutions with different concentrations. **b** Migration of B16F10 and 4T1 cells cultured with JM2 solutions with different concentrations. **c** Invasion of B16F10 and 4T1 cells cultured with JM2 solutions with different concentrations. **d** Q-RT-PCR analysis of MMP2 and MMP9 mRNA in B16F10 and 4T1 cells. * *p* < 0.05 and **p < 0.01 when the data was compared with the Control group.

Figure 3a shows the migration of the tumor cells into the scratched area created in the experiments. Compared with the control group, JM2 solutions could significantly suppress the migration of these tumor cells into the scratched area. In addition, as compared to the control group, fewer B16F10 or 4T1 cells cultured with JM2 solutions migrated through the Transwell chamber (Fig. 3b). Figure 3c further shows that JM2 significantly inhibited the invasion of the tumor cells as fewer cells cultured with JM2 solutions migrated through the Matrigel-coated Transwell chamber as compared with the control group. These results indicate that JM2 can inhibit migration and invasion of B16F10 and 4T1 cells. Matrix metalloproteinase (MMP) such as MMP2 and MMP9, can degrade various protein components in the extracellular matrix and provides favorable conditions for cell migration and invasion [19, 20]. Figure 3d presents the gene expressions of MMP2 and MMP9 in B16F10 and 4T1 cells cultured with different media. Obviously, the addition of JM2 in the cell culture medium significantly suppressed the gene expressions of MMP2 and MMP9 in both types of tumor cells.

### JM2 located inside of tumor cells and regulated mitochondrial distribution

**Fig. 4:**
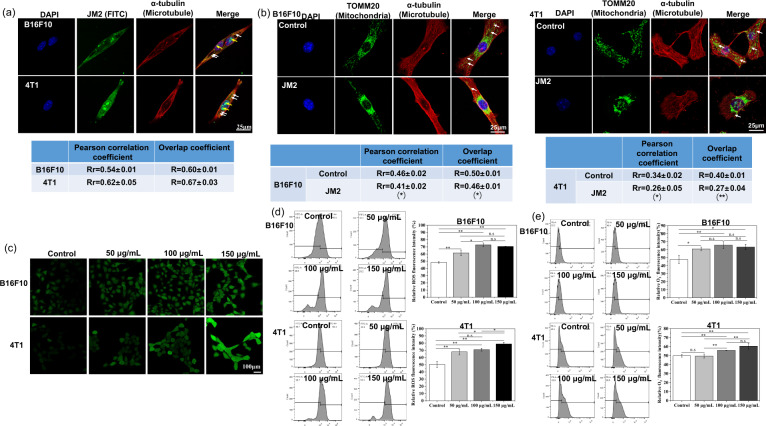
JM2 induced mitochondrial aggregation and production of ROS. **a** JM2 located along with microtubules (white arrows) and accumulated in the nuclei of B16F10 and 4T1 cells (yellow arrows). The Pearson correlation coefficient (PCC) and the Mander‘s overlap coefficient (MOC) represented the degree of colocalization between JM2 and microtubules. **b** JM2 induced the aggregation of mitochondria near the microtubule-organizing center (white arrows). The Pearson correlation coefficient (PCC) and the Mander’s overlap coefficient (MOC) represented the degree of colocalization between mitochondria and microtubules. **c** Fluorescence images of ROS generated in B16F10 and 4T1 cells. **d** Quantified flow data of ROS level in B16F10 and 4T1 cells. **e** Quantified flow data of O2- level in B16F10 and 4T1 cells. *p < 0.05 and **p < 0.01 when the data was compared with the Control group.

Figure 4a shows that JM2 exhibited green fluorescence and microtubules were stained in red fluorescence. Basically, JM2 could be observed in B16F10 and 4T1 cells. Especially, JM2 presented along with the orientation of microtubule distribution (white arrows), which may be caused by the combination of JM2 and microtubules. For B16f10 cells, the Pearson correlation coefficient (PCC) and the Mander’s overlap coefficient (MOC) was Rr = 0.54 ± 0.01 and *R* = 0.60 ± 0.01. The PCC and the MOC was Rr = 0.60 ± 0.01 and *R* = 0.67 ± 0.03 in 4T1 cells, respectively. In addition, it is obvious that abundant JM2 accumulated in the nuclei of the cells (yellow arrows), which may affect synthesis of the chromatin in the nuclei.

In Fig. 4b, more mitochondria could be observed in the microtubule-organizing center (near nuclei) of the cells in JM2 group than that of the cells in control group. In addition, part of the mitochondria could be found to be co-localized with the microtubules (white arrows) in cells, but there were fewer mitochondria co-localized with the microtubules in the cells treated with JM2 as compared to that in the cells in control group. Specifically, for B16F10 cells, the PCC and the MOC was Rr = 0.46 ± 0.02 and *R* = 0.50 ± 0.01 in the control group, the PCC and the MOC were decreased to Rr = 0.41 ± 0.02 and *R* = 0.46 ± 0.01 after the cells were treated with JM2. In 4T1 cells, the PCC and the MOC was Rr = 0.34 ± 0.02 and *R* = 0.40 ± 0.01 in the control group. After the cells were treated with JM2, the PCC and the MOC dropped to Rr = 0.26 ± 0.05 and *R* = 0.27 ± 0.04, respectively. These results suggested that JM2 could regulate the positioning of mitochondria in tumor cells and induce the concentration of mitochondria near the microtubule-organizing center.

### JM2 increased intracellular ROS level and induced mitochondrial dysfunction

As shown in Fig. 4c, the cells cultured with JM2 solutions showed much brighter green fluorescence as compared to those in control group. In addition, flow cytometry was applied to quantitatively analyze the ROS level. The ROS levels in tumor cells were detected and the ROS level of control group was defined as about 50%. It can be seen from Fig. 4d that the ROS levels of B16F10 or 4T1 cells treated with JM2 solutions increased. In addition, the mitochondrial superoxide generation was examined by using MitoSOX Red. In Fig. 4e, the mitochondrial superoxide level was defined as about 50%, and it can be seen that the mitochondrial superoxide levels of B16F10 or 4T1 cells treated with JM2 solutions increased. These results indicated that JM2 treatment could increase the ROS levels in the tumor cells.

**Fig. 5:**
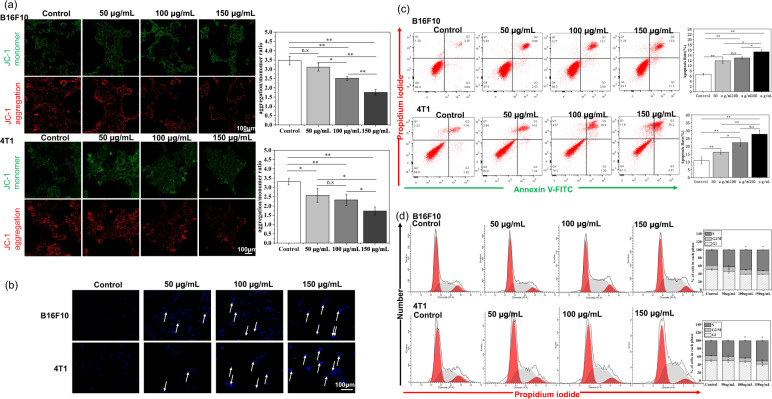
JM2 induced apoptosis in the path of mitochondrion and cell cycle arrest. **a** Mitochondrial membrane potential detection of B16F10 and 4T1 cells cultured with JM2 solutions with different concentrations. The change of aggregation/monomer ratio represent the change of mitochondrial membrane potential. **b** Apoptotic cells exhibited nuclear chromatin aggregation after being treated with JM2 solutions with different concentrations (white arrows). **c** Apoptotic effects and quantitative analysis of B16F10 and 4T1 cells cultured with JM2 solutions with different concentrations. **d** Cell cycle analysis of B16F10 and 4T1 cells cultured with JM2 solutions with different concentrations. *p < 0.05 and **p < 0.01 when the data was compared with the Control group.

Since excessive ROS levels may cause mitochondrial dysfunction, mitochondrial membrane potential detection assay was performed. In Fig. 5a, the red fluorescent JC-1 aggregates indicate high mitochondrial membrane potential, while the green fluorescent JC-1 monomer indicates low mitochondrial membrane potential. When B16F10 and 4T1 cells were cultured with normal culture medium, the cells showed dark green fluorescence and bright red fluorescence. After the cells were cultured with JM2 solutions, the green fluorescence began to brighten, and the red fluorescence began to darken. As the concentration of JM2 solutions increased, the green fluorescence became brighter while the red fluorescence became darker. It can be seen from the statistical graphs that as the concentration of the JM2 solution increased, the aggregation/monomer ratio gradually decreased, which indicated that the membrane potential of the cells gradually decreased both in B16F10 and 4T1 cells. The result suggested that JM2 could decrease the cell mitochondrial membrane potential, which may cause cell apoptosis.

### JM2 led to apoptosis and cell cycle arrest in tumor cells

Chromatin shrinks when tumor cells undergo apoptosis. The nucleus of tumor cells was stained with Hoechst 33258 and the images of the nucleus of tumor cells are shown in Fig. 5b. It can be seen that the nucleus of normal cells appeared normal blue color (control). However, after the B16F10 and 4T1 cells were cultured with JM2 solutions, some of the cell nucleus of the tumor cells showed enhanced blue fluorescence (white arrows) as compared to those of the cells cultured with normal medium.

To quantitatively evaluate the apoptotic effects of JM2 on tumor cells, Annexin V-FITC apoptosis assay was performed to determine the percentage of cell apoptosis. As shown in Fig. 5c, after the B16F10 cells or 4T1 cells were cultured with JM2 solutions, the percentage of cell apoptosis was significantly higher than that of B16F10 or 4T1 cells cultured with normal culture medium (control). Therefore, JM2 solutions led to tumor cell apoptosis and the proportions of apoptotic B16F10 and 4T1 cells were increased markedly in a dose-dependent manner.

**Fig. 6:**
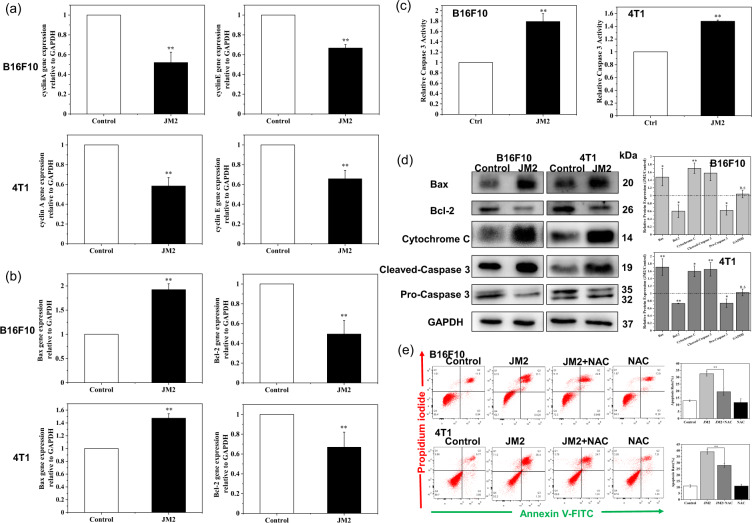
JM2 regulated the expression of genes and proteins related to cell cycle and apoptosis. **a** Q-RT-PCR analysis of cyclin A and cyclin E mRNA in B16F10 and 4T1 cells. **b** Q-RT-PCR analysis of Bax and Bcl-2 mRNA in B16F10 and 4T1 cells. **c** Caspase 3 activity analysis of B16F10 and 4T1 cells cultured with JM2 solutions. **d** Expression of apoptosis-related proteins in B16F10 and 4T1 cells cultured with JM2 solutions. **e** Apoptotic effects and quantitative analysis of B16F10 and 4T1 cells cultured with different culture media. *p < 0.05 and **p < 0.01 when the data was compared with the Control group.

In addition, as displayed in Fig. 5d, B16F10 and 4T1 cells incubated with JM2 solutions showed a prominent S phase arrest as compared with the control group. Furthermore, we found that the gene expression of cyclin A and cyclin E was changed dramatically in B16F10 and 4T1 cells treated with JM2 solutions. As shown in Fig. 6a, compared with the control group, JM2 solution significantly inhibited the gene expressions of cyclin A and cyclin E in both types of tumor cells.

### JM2 induced apoptosis of B16F10 and 4T1 tumor cells via ROS-dependent mitochondrial apoptosis pathway

The generation of ROS plays an important role in regulating DNA damage and apoptosis [21]. Since JM2 could increase the ROS level in tumor cells and induce tumor cell apoptosis, the relationship between cells apoptosis and ROS levels were studied. The gene expression of Bax and Bcl-2 in B16F10 and 4T1 cells was first explored by Q-RT-PCR experiment and the results are shown in Fig. 6b. The gene expression of Bax in B16F10 and 4T1 cells cultured with JM2 solution increased than that of cells cultured with normal culture medium. In contrast, the gene expression of Bcl-2 in B16F10 and 4T1 cells cultured with JM2 solution decreased as compared to that of cells cultured with normal culture medium. It can be seen from the above results that JM2 upregulated the gene expression of the pro-apoptotic protein Bax but downregulated the gene expression of the anti-apoptotic protein Bcl-2 in B16F10 and 4T1 cells. Then, the relative activity of Caspase 3 was calculated and the results are shown in Fig. 6c. After the cells were incubated with JM2 solution, the Caspase 3 activity of B16F10 and 4T1 cells increased.

Moreover, the expressions of the mitochondrial apoptosis-related proteins were significantly changed in B16F10 and 4T1 cells after JM2 treatment. As displayed in Fig. 6d, the expression of pro-apoptotic protein Bax increased in the B16F10 and 4T1 cells treated with JM2 as compared to that in the cells cultured with normal medium. In contrast, the expression of anti-apoptotic protein Bcl-2 decreased in the B16F10 and 4T1 cells treated with JM2 as compared to that in the cells cultured with normal medium. In addition, cytochrome C released into the cytoplasm was increased in B16F10 and 4T1 cells treated with JM2. Additionally, Cleaved-Caspase 3 protein was highly expressed in B16F10 and 4T1 cells after JM2 treatment. Thus, after tumor cells were incubated with JM2 solution, the activated Caspase 3 that mediated apoptosis in the cells increased. Correspondingly, the protein of inactive Pro-Caspase 3 in the cell was reduced. We found that the expression of Pro-Caspase 3 protein in B16F10 and 4T1 cells treated with JM2 was decreased than that in the cells cultured with normal culture medium.The raw data of western blot results were shown in supplementary material.

N-acetyl-L-cysteine (NAC), an antioxidant and ROS scavenger, was used to reduce ROS in B16F10 and 4T1 cells [22]. As shown in Fig. 6e, when the B16F10 or 4T1 cells were cultured with the medium containing JM2 and NAC, the JM2 + NAC combination treatment could significantly attenuate the pro-apoptotic effect of JM2 and reduce the percentage of cell apoptosis. This result demonstrated that ROS level increase played an important role in inducing apoptosis in B16F10 and 4T1 tumor cells by JM2.

### JM2 inhibited tumor recurrence in a tumor resection model

**Fig. 7:**
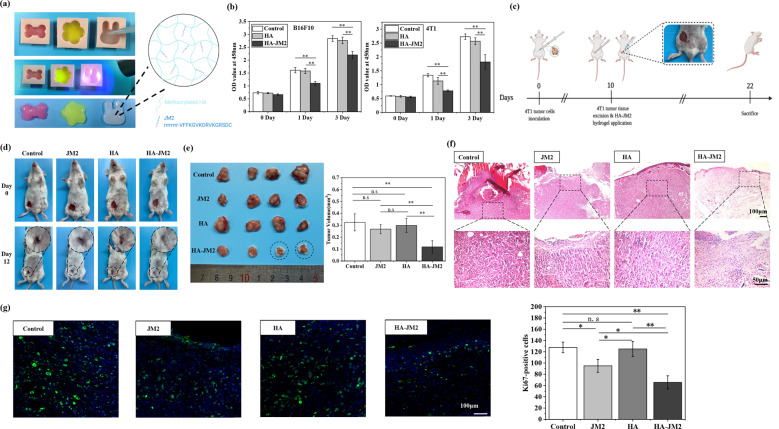
HA-JM2 injectable hydrogel inhibited postsurgical tumor recurrence. **a** Representative pictures of HA-JM2 injectable hydrogel. **b** Cell proliferation evaluation of B16F10 and 4T1 cells cultured with different hydrogels. **c** Schematic illustration of the in vivo tumor recurrence inhibition experimental protocol. **d** Representative gross observation images of post-surgical observation of mice in different groups at day 0 and 12. **e** Representative images and statistical analysis of recurrent tumors in different groups at day 12. **f** H&E staining of recurrent tumors obtained from different groups at day 12. **g** Ki67 immunofluorescence staining (green) and DAPI staining (blue) of recurrent tumors obtained from different groups at day 12. Statistical image displayed the number of Ki67-positive cells. *p < 0.05 and **p < 0.01 when the data was compared with the Control group.

Since JM2 exhibited anti-tumor effects both in vivo and in vitro, a HA-JM2 injectable hydrogel was used to explore the application potential of JM2 in inhibiting tumor recurrence after surgery. In Fig. 7a, the HA-JM2 solution could be crosslinked into hydrogels of different shapes, which proved that the HA-JM2 hydrogel had injectable properties. The inhibitory effect of HA-JM2 hydrogel on the proliferation of B16F10 and 4T1 cells was first explored and the results are shown in Fig. 7b. For B16F10 or 4T1 cells, after being cultured for 24 h, the proliferation inhibition rates of B16F10 and 4T1 cells cultured with HA-JM2 hydrogel were 32% and 42%, respectively. After being cultured for 3 days, it can be seen that the proliferation inhibition rates of B16F10 and 4T1 cells in the HA-JM2 group were about 23% and 33%, respectively. These results indicated that the HA-JM2 hydrogel had effective in vitro anti-tumor effects.

Then, an in vivo orthotopic model of breast cancer by inoculating 4T1 tumor cells was constructed. After 10 days, most of the tumor tissues were excised and tumor tissue with a volume of 20 mm^3^ was intentionally left at the wound site. JM2 solution, HA hydrogels, and HA-JM2 hydrogels were applied to the wounds, and tumor recurrence was observed (Fig. 7c). The gross observation on tumor recurrence of the mice in the four groups was shown in Fig. 7d. The tumors were excised and quantified, and the results are shown in Fig. 7e. It can be clearly observed that the tumor tissue in the HA-JM2 group was the smallest among all groups, and even no tumor tissue can be seen in some samples treated with HA-JM2 hydrogels (black circle). In addition, HE staining showed that there were abundant tumor cells in the control group, JM2 group, and HA group, and the intercellular substance was tight (Fig. 7f). The tissue samples in the HA-JM2 group had few tumor cells and the intercellular substance was loose. In Fig. 7g, the representative images and statistical analysis with less green fluorescence can be observed in the HA-JM2 group than that in the other three groups, indicating that there are fewer malignantly proliferating cells in the tumor tissues of HA-JM2 group than that of other three groups.

## Discussion

According to 2020 global cancer statistics, the number of new cases of cancer and the burden of cancer death worldwide is mushrooming [23]. Clinically, surgical treatment is the first choice for early solid tumors, but tumor recurrence caused by incomplete resection is still the main challenge for patients’ survival after surgery [7, 24]. With the emergence of multi-drug resistance in tumor cells and the side effects caused by chemotherapy, there is an urgent need to discover innovative drugs with high efficiency and low toxicity for the treatment of cancers. In the current study, for the first time, the possible mechanisms of JM2 inhibiting proliferation and inducing apoptosis of tumor cells were firstly explored and proposed and JM2 as well as HA-JM2 hydrogels were applied it to inhibit tumor growth of post-surgical treatments.

**Fig. 8:**
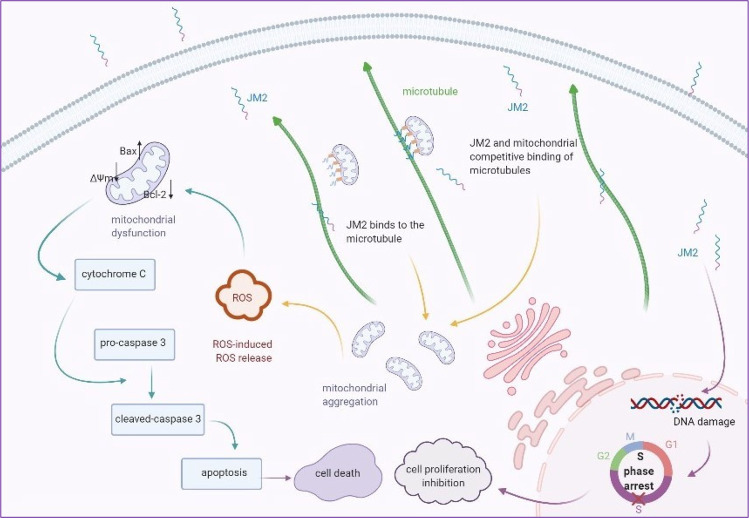
The anti-tumor mechanism of JM2. Illustration of possible molecular mechanism of JM2 applied to tumor therapy.

Apoptosis is a cell death mechanism and can be induced in cells via extrinsic and intrinsic (mitochondrial) pathways [25]. The extrinsic pathway is related to activation of cell surface death receptors and the mitochondria-mediated intrinsic pathway can be activated by different stress conditions [26]. The production of ROS and the release of proteins from the intermembrane space of the mitochondria can lead to the activation of mitochondria-mediated intrinsic apoptosis pathway [27]. Previous studies reported that the process of releasing ROS from one mitochondrion would cause adjacent mitochondria to induce ROS release and constitute a positive feedback mechanism for enhanced ROS generation [14, 18]. Therefore, the excessive ROS generation would cause mitochondrial dysfunction in many mitochondria, leading to potentially significant mitochondria and cell damage. In this study, JM2 entered the cells and bound to microtubules competitively with mitochondria. More JM2 bound to microtubules, fewer mitochondria bound to microtubules to be transported to the periphery of the cells, resulting in the aggregation of mitochondria near the microtubule-organizing center. Whereafter, abundant aggregated mitochondria contributed to arouse RIRR response and induce ROS to accumulate. Excessive ROS generation would lead to mitochondrial dysfunction and an increase in Bax and a decrease in Bcl-2 levels were observed. The imbalance of Bax and Bcl-2 proteins stimulated a decrease of the mitochondrial membrane potential and mitochondrial membrane permeability increased. The subsequent release of Cytochrome C from the mitochondria into the cytosol could trigger Cleaved-Caspase 3 activation, ultimately causing tumor cells apoptosis. The above-described mechanism pathway has been illustrated in Fig. 8.

In addition, cell cycle control represents a major regulatory mechanism for cell proliferation. Once the expressions of genes that control cell division are abnormal, it will lead to uncontrolled cell proliferation, which will further result in tumor development [28]. The results obtained in this study demonstrated that JM2 could induce tumor cells cycle arrested in S phase and JM2 treatment decreased the gene expression of cyclin A and cyclin E. Cyclin A appears in the nucleus in the S phase and participates in the initiation and completion of DNA replication. Cyclin E is needed for cell cycle transition from the G1 phase to the DNA synthesis phase (S phase) [29]. It was speculated that JM2 could regulate the expression of cell cycle-related proteins to stimulate the S phase arrest and consequently inhibited the proliferation of tumor cells (Fig. 8).

Tumor cell migration and invasion refer to the spread of tumor cells from the original area to other sites and continuously grow to form tumor tissues, which is one of the characteristics of malignant tumors. Numerous studies had shown that MMPs were overexpressed in multifarious tumor tissues and were associated with tumor cell migration and invasion. Among them, MMP2 and MMP9 could degrade type IV collagen, elastin, gelatin and other components, directly destroy the extracellular matrix barrier and promote tumor metastasis [20, 30]. In our study, we found that JM2 could suppress migration and invasion of B16F10 and 4T1 cells as well as inhibit the expression of MMP2 and MMP9 in B16F10 and 4T1 cells. Therefore, it was speculated that JM2 could inhibit the expression of MMP2 and MMP9 and consequently inhibited the migration and invasion of tumor cells. As for whether the JM2-inhibited tumor cell migration and invasion involve other signaling pathways, it will be studied in the future.

## Conclusions

In this study, JM2 was demonstrated to be able to inhibit tumor growth in post-surgery treatment. Mechanism study showed that JM2 could effectively inhibit the proliferation of B16F10 and 4T1 cells by decreasing the expressing of cell cycle-related proteins cyclin A and cyclin E to cause cell cycle S phase arrest. Meanwhile, JM2 could inhibit the migration and invasion of B16F10 and 4T1 cells by suppressing the expression of MMP2 and MMP9. It was revealed that JM2 could lead to mitochondria aggregation and induce RIRR response and excessive ROS and mitochondrial superoxide generation induced mitochondria-mediated cell apoptosis. In the mouse model of in situ resection of breast tumor, HA-JM2 hydrogel showed significant anti-tumor activity and inhibited breast tumor recurrence.

## Materials and methods

### In vivo anti-tumor assay

All animal experiment protocols were approved by the Institutional Review Committee of Shanghai Jiao Tong University, School of Biomedical Engineering. The approval number is 2019008. In this study, ten BALB/c female mice aged 8 weeks were selected and randomly divided into two groups. JM2 powders were dissolved in PBS to obtain the JM2 intratumoral injection solution with a concentration of 2.5 mg mL^−1^. 4T1 cells were collected, resuspended in PBS and 2 × 10^5^ cells were subcutaneously inoculated into the right-back of each mouse. After 4 days, the mice in the control group and JM2 group were administrated with 50 µL PBS or JM2 (2.5 mg kg^−1^) every 3 days. Then, the body weight of the mice and tumors were measured every 2 days. The relative tumor volumes were calculated according to the formula *V* [mm^3^] = *ab*^2^/2, where *a* and *b* are the length and width of the tumors, respectively.

On day 14, all mice were sacrificed, and the tumor tissues were collected and photographed before they were fixed with 4% paraformaldehyde (PFA), embedded in paraffin, and sectioned into slices. Part of tumor tissue section samples was dewaxed and stained with hematoxylin and eosin (Yeasen, China). The stained slices were observed with an optical microscope (Leica DMI 3000B, Germany) and photographed by using a CCD camera connected to the microscope.

In addition, proliferation of cells in tumor tissues were detected by staining the tissue sections with a cellular proliferation marker, Ki67. Briefly, hydrated slices were antigen heat repaired by 0.01 M heated sodium citrate buffer. Then, the slices were incubated with 0.3% H_2_O_2_/methanol for 30 min before they were incubated with 5% bovine serum albumin (BSA, Sigma, USA)-PBS solution for 1 h and incubated with primary antibody of rabbit anti-Ki67 (1:200, Abcam, UK) overnight. After that, the slices were incubated with HRP-conjugated goat anti-rabbit secondary antibody (Abcam, UK) and then incubated with the DAB solution in a DAB substrate kit (Abcam, UK). The tissue sections were further stained with hematoxylin before they were observed under the optical microscope.

Moreover, apoptotic cells in tumor tissues were detected by TUNEL apoptosis detection kit (Yeasen, China) according to the instruction. Briefly, the hydrated slices were incubated with 20 µg mL^−1^ Proteinase K-PBS solution before they were incubated with equilibration buffer. Meanwhile, 50 µL system TdT incubation buffer was prepared according to the instruction. After that, the equilibration buffer was replaced by TdT incubation buffer and incubated at 37 °C. The nuclei of the cells were stained with 5 µg mL^−1^ 4,6-diamidino-2-phenylindole (DAPI). Finally, the TUNEL-stained slices were observed and photographed with a camera (Leica DFC 420 C) connected to a confocal laser scanning microscope (Leica SP5, Germany).

### Cell culture

B16F10 melanoma cell lines were purchased from Zhong Qiao Xin Zhou Biotechnology Co., Ltd (Shanghai, China) and 4T1 cell lines were acquired from the Shanghai Institute of Cell Biology of the Chinese Academy of Sciences (Shanghai, China). B16F10 cells were cultured with RPMI-1640 culture medium (ZQ-201, Zhong Qiao Xin Zhou Biotechnology Co., Ltd, Shanghai, China) and 4T1 cells were cultured with Dulbecco’s Modified Eagle Medium (DMEM, Gibco) containing 10% fetal bovine serum (FBS) and 1% penicillin–streptomycin (P/S).

### JM2 mimic peptide preparation

The JM2 mimic peptide was designed based on our previous study, containing a poly-d-arginine internalization vector, a Cx43 microtubule-binding domain, and a cysteine residue at the C-terminal end (JM2, rrrrrrrr-VFFKGVKDRVKGRSDC). In order to observe the position of JM2 in cells, the JM2 mimic peptide was labeled with FITC fluorescein on its N-terminal (FITC-JM2, FITC-rrrrrrrr-VFFKGVKDRVKGRSDC). The JM2 and FITC-JM2 mimic peptide was customized and synthesized by GL Biochem Ltd (Shanghai, China).

### Effects of JM2 on proliferation and cytotoxicity of tumor cells

#### Cell proliferation assay

Before all experiments, JM2 solutions with different concentrations were prepared. JM2 powders were dissolved in the normal cell culture medium (RPMI-1640 or DMEM) to obtain JM2 solutions with concentrations of 50, 100, 150, 200 µg mL^−1^, respectively. Then, the JM2 solutions were filtered and sterilized with 0.22 µm filters (Millipore, USA). In subsequent in vitro cell experiments, JM2 solutions with different concentrations were prepared by this way unless otherwise noted. B16F10 or 4T1 cells were seeded in 48-well plates at a density of 1 × 10^4^ cells per well and cultured in a humidified 5% CO_2_ incubator at 37 °C. After 12 h, the culture medium was discarded and cells were washed with PBS. Then, 200 µL fresh normal cell culture medium containing 20 µL Cell Counting Kit-8 (CCK-8, Beyotime, China) solution was added into each well and the cells were further cultured for 1.5 h. The absorbance of cells at 450 nm was measured by a microplate reader (Synergy 2, Bio-TEK). Then, the cells were washed with PBS and cultured with JM2 solutions with concentrations of 50, 100, 150, 200 µg mL^−1^ for another 1 and 3 days. RPMI-1640 and DMEM was used as the culture medium in the control group for B16F10 and 4T1 cells, respectively. At predetermined culture time points, the culture medium was discarded and cells were cultured with normal culture medium containing CCK-8 for 1.5 h. The absorbance of cells at 450 nm was measured by a microplate reader. Finally, the cell proliferation inhibition rate was calculated by the formula CV = (1 − OD_Experimental group_/OD_Control group_) × 100%. Three independent experiments were carried out for validation.

#### Lactate dehydrogenase (LDH) release assay

In this experiment, JM2 solutions with different concentrations were prepared by using culture medium containing 1% FBS. B16F10 or 4T1 cells were seeded in 96-well plates at a density of 1 × 10^4^ cells per well and cultured for 12 h. Then, the culture medium was discarded and replaced by JM2 solutions with concentrations of 50, 100, 150, 200 µg mL^−1^. RPMI-1640 and DMEM containing 1% FBS was used as the culture medium in the control group for B16F10 and 4T1 cells, respectively. Wells with no cells but containing culture medium were considered as blank group. After 24 h, the plate was centrifuged at 400 × *g* for 5 min, and 120 μL supernatants per well were transferred to wells in another new 96-well plate. The LDH detection working solution was prepared according to the manufacturer’s instructions. Sixty microliters LDH detection working solution was added to each well and the plate was incubated at room temperature for 30 min. Then, the absorbance of the mixture at 490 nm was measured by a microplate reader. Finally, relative LDH release (RLR) was calculated by the formula RLR = (OD_Experimental group_ – OD_Blank group_)/(OD_Control group_ – OD_Blank group_). Three independent experiments were carried out for validation.

#### Colony formation assay

B16F10 or 4T1 cells were seeded in 6-well plates at a density of 1.5 × 10^5^ cells per well and cultured for 12 h. Then, the culture medium was replaced by JM2 solutions with concentrations of 50, 100, 150, 200 µg mL^−1^. After 24 h, the cells treated with JM2 solutions with different concentrations were detached with trypsin and collected separately. The collected cells were seeded in 6-well plates at a density of 200 cells per well and cultured for 10 days. Cells treated without JM2 solutions were used as control group. Finally, the cells were fixed in 4% PFA and stained with crystal violet. The cells in the plates were photographed and the colonies formed were counted. According to the cell proliferation assay, LDH release assay and colony formation assay, the effective concentrations of JM2 solutions can be determined and used for subsequent in vitro experiments. Three independent experiments were carried out for validation.

### Effects of JM2 on migration and invasion of tumor cells

#### In vitro scratch assay

In this experiment, JM2 solutions with different concentrations of 50, 100, and 150 µg mL^−1^ were prepared by using serum-free culture medium or low serum culture medium (serum-free RPMI-1640 or 1% FBS DMEM). 4T1 cells were sensitive in serum-free medium and easily fall off the bottom of the well. B16F10 or 4T1 cells were seeded in 24-well plates at a density of 5 × 10^4^ cells per well and cultured for 24 h. Then, cell culture medium was discarded and the monolayer of cells was scratched vertically in a straight line by using 200 µL pipette tips on the bottom of each well. Then, the cells were washed twice with PBS and cultured with JM2 solutions with different concentrations for 12 and 24 h. Serum-free RPMI-1640 and 1% FBS DMEM was used as the medium for B16F10 and 4T1 cells in control group, respectively. At the predetermined time, the scratches were photographed by using a digital camera (Leica DFC 420C) connected with an inverted microscope (Leica DMI 3000B, Germany).

#### In vitro transwell migration/invasion assay

The migration ability of B16F10 and 4T1 cells was assessed by using a Transwell chamber assay. B16F10 and 4T1 cells were collected and resuspended in serum-free RPMI-1640 or serum-free DMEM medium, respectively, at a density of 3 × 10^5^ cells mL^−1^. One hundred microliters of cell suspension was added to the upper of the Transwell chamber (pore size 8 μm, Corning, USA), and 600 μL of JM2 solutions with different concentrations of 50, 100, and 150 µg mL^−1^ were loaded into the lower chambers in 24-well plates. RMPI-1640 and DMEM culture medium were used as the control group for B16F10 and 4T1 cells, respectively. After 6 h, the culture medium in the upper chamber was discarded and the cells in the upper chamber were wiped off. The cells migrating through the polycarbonate membrane were fixed with 4% PFA for 10 min, followed by staining with crystal violet for 20 min. Finally, the cells were washed twice with PBS. The cells passing through the polycarbonate membrane were photographed and counted by using a digital camera connected with an inverted microscope. Three independent experiments were carried out for validation.

The invasive ability of B16F10 and 4T1 cells was evaluated by using a Matrigel-assisted Transwell chamber assay. Matrigel was used to imitate extracellular matrix in vivo. Tumor cells need to secrete MMPs to degrade the Matrigel before they pass through the polycarbonate membrane of Transwell chamber. Therefore, the number of cells passing through the membrane reflect the invasion ability of tumor cells. In this experiment, JM2 solutions with different concentrations of 50, 100, and 150 µg mL^−1^ were prepared by using serum-free culture medium or low serum culture medium (serum-free RPMI-1640 or 1% FBS DMEM). In details, Matrigel (BD Biocoat, 356234) was diluted with ice-cold serum-free RPMI-1640 and serum-free DMEM at a ratio of 1:8 and 100 μL of diluted Matrigel was added to the upper chamber of the Transwell in a 24-well plate. All of the above steps were conducted on ice. Then, the 24-well plate was placed at 37 °C and incubated overnight to convert the Matrigel solution into a gel. B16F10 and 4T1 cells were collected and resuspended in serum-free or low serum JM2 solutions with different concentrations at the cell density of 1 × 10^6^ cells mL^−1^. Then, 100 μL of the cell suspension was added into the upper chamber of Transwell and 600 μL of RPMI-1640 or DMEM was added to the lower chamber of the 24-well plate. For the control group, 100 μL of the cell suspension resuspended in serum-free RPMI-1640 or DMEM was added to the upper chamber and 600 μL of RPMI-1640 or DMEM was added to the lower chamber. After 24 h, the culture medium and Matrigel in the upper chamber were discarded and the cells in the upper chamber were removed with a cotton swab. The cells migrating through the polycarbonate membrane were fixed with 4% PFA and stained with crystal violet. Finally, the cells were photographed and counted by using a digital camera connected with an inverted microscope. Three independent experiments were carried out for validation.

### Intracellular localization of JM2 and mitochondria

#### Intracellular localization of JM2

In this experiment, FITC-JM2 solution with a concentration of 150 µg mL^−1^ was prepared by dissolving FITC-JM2 powder in RPMI-1640 or DMEM culture medium. B16F10 or 4T1 cells were seeded in glass bottom cell dish (Nest, 801002, China) at a density of 2 × 10^4^ cells per dish and cultured for 24 h. Then, the culture medium was replaced by 150 µg mL^−1^ FITC-JM2 solution. After 4 h, the culture medium was discarded and the cells were washed twice with PBS. Then, the cells were fixed with 4% PFA for 10 min and permeabilized with 0.3% triton-x100 for 5 min. After that, the cells were blocked with 1% BSA-PBS solution at 37 °C. The cells were incubated with mouse anti-α-tubulin (Sigma, USA) primary antibody (diluted with 0.5% BSA-PBS solution at the ratio of 1:300) and kept at 4 °C overnight. The cells were then washed with PBS for three times and stained with Alexa 594 goat anti-mouse IgG (Invitrogen, USA) (diluted with 0.5% BSA-PBS at the ratio of 1:1000). At the end of the incubation, the cells were washed with PBS before the nuclei of the cells were stained with 5 µg mL^−1^ DAPI solution. Finally, the stained cells were observed and photographed with a camera connected to a confocal laser scanning microscope. The PCC and the MOC were analyzed to quantify the degree of colocalization between JM2 and microtubules. Three independent experiments were carried out for validation.

#### Intracellular localization of mitochondria

B16F10 and 4T1 cells were seeded on coverslips fitted in 24-well plates at a density of 3 × 10^4^ cells per well and cultured for 24 h. Then, the culture medium was replaced by 150 µg mL^−1^ JM2 solution. The cells cultured with RPMI-1640 or DMEM were considered as the control group for B16F10 and 4T1 cells, respectively. After 4 h, the culture medium was discarded and the cells were washed twice with PBS. Co-immunofluorescence staining of TOMM20 and α-tubulin was performed according to the method in *Intracellular localization of JM2*. The primary antibody of TOMM20 rabbit monoclonal antibody (Beyotime, China) and mouse anti-α-tubulin (Sigma, USA) were diluted with 0.5% BSA-PBS solution at the ratio of 1:300. The cells were then incubated with mixed primary antibody solution and kept at 4 °C overnight. After that, the cells were stained with a mixture of Alexa 488 goat anti-rabbit IgG (Invitrogen, USA) and Alexa 594 goat anti-mouse IgG (Invitrogen, USA) diluted at the ratio of 1:1000, and the nuclei of the cells were stained with 5 µg mL^−1^ DAPI solution. Finally, the stained cells were observed and photographed with a camera connected to a confocal laser scanning microscope. The PCC and the MOC were analyzed to quantify the degree of colocalization between mitochondria and microtubules. Three independent experiments were carried out for validation.

### Assessments of intracellular ROS and mitochondrial superoxide production

Intracellular ROS and mitochondrial superoxide were assessed by using H_2_O_2_-sensitive probe, 2,7-Dichlorodi-hydrofluorescein diacetate (DCFH-DA) or mitochondrial superoxide probe, MitoSOX red. B16F10 and 4T1 cells were seeded on coverslips fitted in 24-well plates at a density of 3 × 10^4^ cells per well and cultured for 12 h. Then, the culture medium was replaced by JM2 solutions with concentrations of 50, 100 and 150 µg mL^−1^. The cells cultured with RPMI-1640 or DMEM were considered as the control groups. DCFH-DA or MitoSOX red was diluted with serum-free RPMI-1640 or DMEM culture medium at a ratio of 1:1000 to a final concentration of 10 μmol L^−1^ or 2 μmol L^−1^. After 24 h, the culture medium was discarded and 250 μL diluted DCFH-DA or MitoSOX red solution was added to each well. The plate was incubated at 37 °C for 20 min before the cells were washed three times with serum-free cell culture medium. Then, the cells treated with DCFH-DA were immediately observed and photographed with a camera connected to a confocal laser scanning microscope. In addition, B16F10 or 4T1 cells were seeded in 6-well plates at a density of 1.5 × 10^5^ cells per well and cultured for 12 h. Then, same cultures and treatments to the above procedures were applied to the cells before they were detached with trypsin and resuspended in 500 μL PBS for flow cytometric analysis by using a flow cytometer (FACS AriaII, BD). Three independent experiments were carried out for validation.

### Determination of cell apoptosis

#### Mitochondrial membrane potential detection

The change of mitochondrial membrane potential is a sign of mitochondrial dysfunction, and the decrease of mitochondrial membrane potential is a landmark event in the early stage of apoptosis [31]. A mitochondrial membrane potential assay kit with JC-1 (Beyotime, China) was used to monitor the changes of mitochondrial membrane potential. When the mitochondrial membrane potential is relatively normal, JC-1 gathers in the matrix of the mitochondria to form red-fluorescencent aggregates. When the mitochondrial membrane potential is decreased, JC-1 cannot accumulate in the matrix of the mitochondria and exists as green-fluorescencent monomer. B16F10 or 4T1 cells were seeded in glass bottom cell dish at a density of 2 × 10^4^ cells per dish and cultured for 12 h. Then, the culture medium was replaced by JM2 solutions with concentrations of 50, 100 and 150 µg mL^−1^. Meanwhile, JC-1 staining working solution was prepared according to the instruction. After 24 h, the culture medium was replaced by 1 mL fresh culture medium and 1 mL JC-1 staining working solution. The cells were further incubated at 37 °C for 20 min and JC-1 staining buffer was prepared according to the instruction during the incubation period. The JC-1 staining working solution was discarded and the cells were washed twice with JC-1 staining buffer. At the end, the cells were immediately observed and photographed with a camera connected to a confocal laser scanning microscope. Image J software was used to quantify the red and green fluorescence, and the ratios of red/green (aggregation/monomer) were calculated. Three independent experiments were carried out for validation.

#### Cell apoptosis assay

B16F10 and 4T1 cells were seeded in 12-well plates at a density of 5 × 10^4^ cells per well and cultured for 12 h. Then, the culture medium was discarded and the cells were cultured with JM2 solutions with concentrations of 50, 100, 150 µg mL^−1^ for 24 h. B16F10 and 4T1 cells cultured with RPMI-1640 or DMEM were regarded as the control groups. After 24 h, the cell culture medium was discarded and the cells were washed with PBS. 300 µL of Hoechst 33258 staining solution was added and the cells were incubated at room temperature for 5 min. Then, the staining solution was discarded and the cells were washed with PBS. At the end, the cells were immediately observed and photographed by using a digital camera connected with an upright microscope.

Meanwhile, flow cytometry was used to detect cell apoptosis. B16F10 and 4T1 cells were seeded in 6-well plates at a density of 1.5 × 10^5^ cells per well and cultured for 12 h. Then, the culture medium was discarded and the cells were cultured with JM2 solutions with concentrations of 50, 100, 150 µg mL^−1^ for 24 h. B16F10 and 4T1 cells cultured with RPMI-1640 or DMEM were regarded as the control group. After 24 h, the cell culture medium and the cells were collected. The cells were resuspended for staining according to the instruction in Annexin V-FITC/PI Apoptosis Detection Kit (Yeasen, China). Briefly, the cells were resuspended in Annexin-FITC binding buffer and added with 5 μL Annexin V-FITC and 10 μL PI staining solution. Then, the cells were incubated at room temperature in dark for 20 min. Finally, the samples were analyzed by using a flow cytometer. Three independent experiments were carried out for validation.

In addition, in order to verify the effect of ROS generated in cells on cell apoptosis, the antioxidant NAC was used in this experiment. B16F10 or 4T1 cells were seeded in 6-well plates at a density of 1.5 × 10^5^ cells per well and cultured for 12 h. The cells were divided into four groups according to different cell culture medium, including control group (RPMI-1640 or DMEM), JM2 group (100 µg mL^−1^ JM2 solution), JM2 + NAC group (100 µg mL^−1^ JM2 solution and 5 mM NAC), NAC group (5 mM NAC). For the JM2 + NAC group and the NAC group, 5 mM NAC was added 2 h in advance. Subsequently, 100 µg mL^−1^ JM2 solution was added to the JM2 group and the JM2 + NAC group. All cells were further cultured for 24 h. Then, the cell culture medium and the cells were collected. The collected cells were stained via the aforementioned staining method by using Annexin V-FITC/PI Apoptosis Detection Kit. Finally, the samples were placed on ice and analyzed by using a flow cytometer. Three independent experiments were carried out for validation.

#### Caspase 3 activity assay

It is generally believed that Caspase 3 is the most important terminal shearing enzyme in the process of cell apoptosis, and it plays an irreplaceable role in cell apoptosis [32]. Caspase 3 activity assay kit (Beyotime, China) was used to detect the activity of the Caspase 3 enzymes in B16F10 and 4T1 cells. Caspase 3 can catalyze the substrate Ac-DEVD-pNA to produce yellow pNA, so the activity of Caspase 3 can be detected by measuring the absorbance. B16F10 and 4T1 cells were cultured according to the method in *Cell apoptosis assay* and the cells were incubated with 100 µg mL^−1^ JM2 solution. After 24 h of incubation, the cell culture medium and the cells were collected and resuspended in 100 µL lysis buffer and placed on ice for 15 min. Subsequently, the cells were centrifuged at 20,000 × *g* for 15 min to obtain the supernatant for Caspase 3 enzyme activity determination. According to the instructions in the Caspase 3 activity assay kit, 40 µL detection buffer, 50 µL sample and 10 µL Ac-DEVD-PNA were added successively to each well of the 96-well plate. Then, the plate was placed in a 37 °C incubator and incubated for 120 min. The absorbance of mixtures at 405 nm was measured by a microplate reader. The Bradford (Beyotime, China) method was used to detect the total protein concentration of the samples. Coomassie brilliant blue combines with arginine in protein and turns into blue after being combined in an acidic medium, and the color change is directly proportional to the protein concentration. In brief, 5 µL samples and 250 µL Coomassie brilliant blue staining solution were added to the wells in 96-well plate. The absorbance of mixtures at 595 nm was measured by a microplate reader. The concentration of Caspase 3 and the concentration of total protein in supernatant were calculated. The concentration of Caspase 3 per mg protein in the control group and the JM2 experimental group was calculated. The relative activity of Caspase 3 was presented as the multiple of the caspase activity of the JM2 experimental group relative to the control group. Three independent experiments were carried out for validation.

### Cell cycle detection assay

B16F10 and 4T1 cells were seeded in 6-well plates at a density of 1.5 × 10^5^ cells per well and cultured for 12 h. The culture medium was then replaced by serum-free RPMI-1640 or serum-free DMEM. After 24 h, the cells were treated according to the method in *Cell apoptosis assay*. The collected cells were fixed in a mixed solution containing 300 µL ice-cold PBS and 700 µL cold ethanol at 4 °C for 2 h. At the same time, the staining working solution was prepared according to the instruction of the Cell Cycle and Apoptosis Analysis Kit (Beyotime, China). The cells were resuspended in the staining working solution and incubated for 30 min in dark. Finally, the cell suspension was filtered with a 300-mesh screen and the DNA content was measured by using a flow cytometer. Three independent experiments were carried out for validation.

### Quantitative real-time polymerase chain reaction (Q-RT-PCR) analysis

In order to detect the effects of JM2 on gene expression of cell migration and invasion-related proteins, cell apoptosis-related proteins, and cell cycle-related proteins, Q-RT-PCR assays were performed. B16F10 and 4T1 cells were seeded and cultured according to the method in *Cell apoptosis assay*. Then, the cells were collected, and the total RNA was extracted with E.Z.N.A. Total RNA kit I (OMEGA, Biotek). The RNA concentration was measured with Nanodrop 1000 reader (Thermo, USA) and cDNA was prepared in the process of reverse transcription by using Hifair^®^ II 1st Strand cDNA Synthesis SuperMix for qPCR (gDNA digester plus) (Yeasen, China). Subsequently, cDNA was diluted with sterilized deionized water at the ratio of 1:10. A 10 μL system was loaded in a 384-well plate including 4.2 μL diluted cDNA mixed with 0.4 μL primer F, 0.4 μL primer R and 5 μL of Hieff^®^ qPCR SYBR Green Master Mix (High Rox) (Yeasen, China). The Glyceraldehyde 3-phosphate dehydrogenase (GAPDH) was used as a housekeeping gene and the sequences of each primer utilized in the experiment were summarized as follows (all primers were purchased from Sangon Biotech Co. Ltd., China). Finally, the mixed solution of cDNA and primers was analyzed via the 7900 real-time PCR system (Applied Biosystems). The results were analyzed by the 2^−ΔΔCt^ method using the SDS 2.4 software. The gene expression was normalized to the gene expression of GAPDH and compared to the control group. Three independent experiments were carried out for validation.

### Sequences

MMP2 (F): ACTTTGAGAAGGATGGCAAGTA

MMP2 (R): CTTCTTATCCCGGTCATAGTCC

MMP9 (F): CAAAGACCTGAAAACCTCCAAC

MMP9 (R): GACTGCTTCTCTCCCATCATC

Bax (F): TTGCCCTCTTCTACTTTGCTAG

Bax (R): CCATGATGGTTCTGATCAGCTC

Bcl-2 (F): GATGACTTCTCTCGTCGCTAC

Bcl-2 (R): GAACTCAAAGAAGGCCACAATC

cyclin A (F): CTGCTAGCTTCGAAGTTTGAAG

cyclin A (R): CATTCTCAGAACCTGCTTCTTG

cyclin E (F): GCACCAGTTTGCTTATGTTACA

cyclin E (R): GGGCCTTCATCATCATCAATTC

GAPDH (F): GATTTGGTCGTATTGGGCG

GAPDH (R): CTGGAAGATGGTGATGG

### Western blot analysis

To ulteriorly explore the effects of JM2 on mitochondria-mediated cell apoptosis-related proteins, western blot assays were performed. B16F10 and 4T1 cells were cultured and treated according to the method in *Cell apoptosis assay*. The collected cells were lysed by using RIPA lysis buffer (Yeasen, China) containing protease inhibitor cocktail (Beyotime, China) and phosphatase inhibitor cocktail (Sigma, USA). The lysates were centrifuged at 12,000 × *g* for 10 min and the supernatants were collected. BCA Protein Quantification Kit (Yeasen, China) was used to quantify the protein in the collected supernatants. The protein samples were mixed with 5× loading buffer and denatured by heating in metal bath for 5 min. Equal amounts of protein samples were separated by 10 or 12% SDS-PAGE gels and transferred to PVDF membranes. After blocked with blocking buffer (Beyotime, China) for 1 h, the membranes were incubated with primary antibodies against Bax (Beyotime, China), Bcl-2 (Beyotime, China), Cytochrome C (Beyotime, China), Cleaved-Caspase 3 (Cell Signaling Technology, USA), Pro-Caspase 3 (Beyotime, China) and GAPDH (Abcam, UK) diluted at the ratio of 1:1000 at 4 °C overnight. Then the membranes were incubated with horseradish peroxidase (HRP) ‐conjugated secondary antibodies diluted at the ratio of 1:1000 for 4 h on ice. The membranes were dropped with BeyoECL Plus solution (Beyotime, China) and photographed with a Tanon-5200 GelCap ECL system (Shanghai, China). Three independent experiments were carried out for validation.

### In vivo tumor recurrence inhibition assay

First of all, freeze-dried JM2 grafted HA were prepared according to our previous research [33]. The freeze-dried JM2 grafted HA was sterilized under UV lamp for 30 min and mixed with photoinitiator LAP (Sigma, USA) at a ratio of 9:1. Then, the mixture was dissolved in PBS. Two hundred microliters mixture solution was injected in plastic molds with different shapes and exposed to the irradiation of UV light (365 nm, Scientz Biotechnology Co., Ltd) for 10 s to crosslinked into HA-JM2 hydrogels. In addition, in order to verify whether the obtained HA-JM2 hydrogels can exert a tumor inhibitory effect, the effect of HA-JM2 hydrogel on the proliferation of B16F10 and 4T1 cells was explored by using a CCK-8 kit. Briefly, B16F10 or 4T1 cells were seeded in 24-well plates at a density of 3 × 10^4^ cells per well and cultured for 12 h. The culture medium was discarded and cells were washed with PBS. 100 μL HA or HA-JM2 solution was added to the upper of the Transwell chamber and exposed to the UV light for 10 s before 600 μL RMPI-1640 or DMEM was loaded into the lower chamber in a 24-well plate. RMPI-1640 and DMEM culture medium was used as the control medium for B16F10 and 4T1 cells, respectively. On days 0, 1, and 3, the culture medium was removed and cells were further cultured with normal culture medium containing CCK-8 for 1.5 h. The absorbance of the cells at 450 nm was measured with a microplate reader and the ability of HA and HA-JM2 hydrogels to inhibit tumor cell proliferation was analyzed.

Then, a mouse model of in situ resection of breast tumor was constructed. Sixteen BALB/c female mice aged 8 weeks were selected and the fourth pair of breasts were injected with 2 × 10^5^ 4T1 cells in situ. After 10 days, these tumor-bearing mice were randomly divided into four groups, including tumor excision treated with nothing (control), tumor excision treated with JM2 solution, tumor excision treated with HA hydrogels, and tumor excision treated with HA-JM2 hydrogels. Tumor tissue with a volume of 20 mm^3^ was intentionally left at the wound sites. According to the divided groups, JM2 solution, HA hydrogels, and HA-JM2 hydrogels were applied to the wounds of each group respectively. During the treatment, the hydrogel dressings were changed on day 3 and 6. On days 0 and 12, the wounds were photographed and all the mice were sacrificed on day 12. Then, the tissues were extracted the fixed with 4% PFA, embedded in paraffin, and sectioned into slices. The slices were dehydrated and used for H&E staining and Ki67 immunofluorescence staining. The H&E staining samples were observed by using an optical microscope and images were taken with a CCD camera connected to the microscope. Ki67 immunofluorescence staining was performed according to the method in In vivo *anti-tumor assay*. The slices were incubated with primary antibody at 4 °C overnight and then incubated with Alexa 488 goat anti-rabbit IgG for 2 h. The nuclei of the cells were stained with 5 µg mL^−1^ DAPI solution and the stained cells were observed and photographed with a camera connected to a confocal laser scanning microscope. Image J software was used to calculate the Ki67-positive cells.

### Statistical analysis

At least three independent experiments were carried out for statistical analysis. All the experimental data were expressed as means ± standard deviation. Figures 3d, 4b, 6–d were used Student’s *t* test to evaluate the significant difference between two groups. Figures 1c, d, 2a, 7b were used two-way analysis of variance (ANOVA) for statistical differences. Figures 2b, c, 3b, c, 4d–f, 5a, c, d, 6e, 7e, g were used one-way ANOVA for statistical differences. PASS 15 software was used to perform power analysis on experimental data to test whether the number of test repeats used in experiments is sufficient. *p < 0.05 and **p < 0.01 indicates statistically significant difference while *p* > 0.05 was considered as no obvious significant difference (n.s).

## Supplementary information

SUPPLEMENTAL MATERIAL

Reproducibility checklist

## Data Availability

All data needed to evaluate the conclusions in the paper are present in the paper. Additional data related to this paper may be requested from the corresponding author.
