# Supplementary material for: Juxtamembrane 2 mimic peptide competitively inhibits mitochondrial trafficking and activates ROS-mediated apoptosis pathway to exert anti-tumor effects
Source: Cell Death Dis. 2022 Mar 24;13(3):264. doi: 10.1038/s41419-022-04639-6 (PMC8948362; doi:10.1038/s41419-022-04639-6)
Supplement: Supplementary file 1 — SUPPLEMENTAL MATERIAL [file 41419_2022_4639_MOESM1_ESM.docx]

**Supplemental Material**

**Supplemental figures**


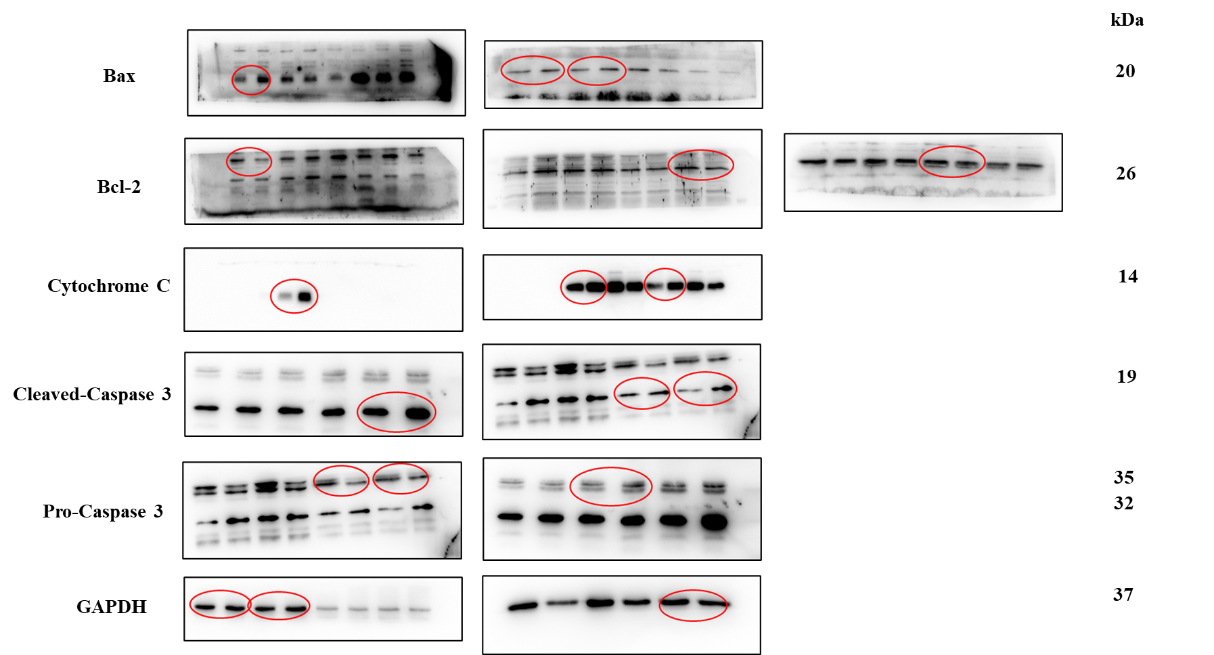


**Figure S1** Original full and uncropped western blots images of apoptosis-related protein expression in B16f10 cells. Red circle represents the results of apoptosis-related protein expression.

**
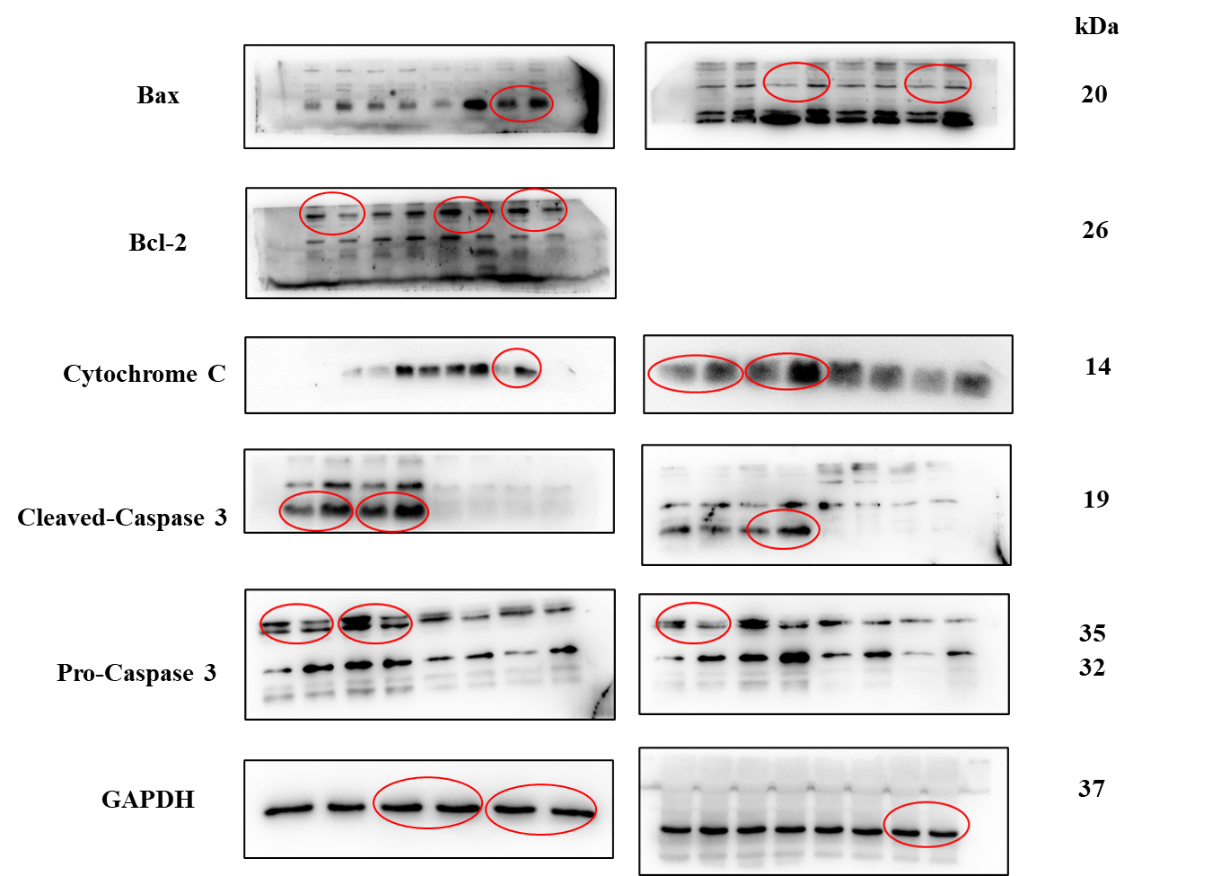
**

**Figure S2** Original full and uncropped western blots images of apoptosis-related protein expression in 4T1 cells. Red circle represents the results of apoptosis-related protein expression.
